# From Bacillus Criminalis to the Legalome: Will Neuromicrobiology Impact 21st Century Criminal Justice?

**DOI:** 10.3390/brainsci15090984

**Published:** 2025-09-13

**Authors:** Alan C. Logan, Barbara Cordell, Suresh D. Pillai, Jake M. Robinson, Susan L. Prescott

**Affiliations:** 1 Nova Institute for Health, Baltimore, MD 21231, USA; susan.prescott@uwa.edu.au; 2 Auto-Brewery Syndrome Information and Research, Carthage, TX 75633, USA; barbaracordell@autobrewery.org; 3 Department of Food Science and Technology, Texas A&M University, College Station, TX 77840, USA; s-pillai@tamu.edu; 4 College of Science and Engineering, Flinders University, Bedford Park, SA 5042, Australia; jake.robinson@flinders.edu.au; 5 The Aerobiome Innovation and Research Hub, Flinders University, Bedford Park, SA 5042, Australia; 6 School of Medicine, University of Western Australia, Perth, WA 6009, Australia; 7 Department of Family and Community Medicine, School of Medicine, University of Maryland, Baltimore, MD 21201, USA

**Keywords:** microbiome, aggression, auto-brewery syndrome, biological criminology, omics, legalome, neuromicrobiology, neurophysiology, forensic neuropsychiatry

## Abstract

The idea that gut microbes or a “bacillus of crime” might promote criminal behavior was popularized in the early 20th century. Today, advances in neuromicrobiology and related omics technologies are lending credibility to the idea. In recent cases of dismissal of driving while intoxicated charges, courts in the United States and Europe have acknowledged that gut microbes can manufacture significant amounts of systemically available ethanol, without a defendant’s awareness. Indeed, emergent research is raising difficult questions for criminal justice systems that depend on prescientific notions of free moral agency. Evidence demonstrates that gut microbes play a role in neurophysiology, influencing cognition and behaviors. This may lead to justice involvement via involuntary intoxication, aggression, anger, irritability, and antisocial behavior. Herein, we discuss these ‘auto-brewery syndrome’ court decisions, arguing that they portend a much larger incorporation of neuromicrobiology and multi-omics science within the criminal justice system. The legalome, which refers to the application of gut microbiome and omics sciences in the context of forensic psychiatry/psychology, will likely play an increasing role in 21st century criminal justice. The legalome concept is bolstered by epidemiology, mechanistic bench science, fecal transplant studies, multi-omics and polygenic research, Mendelian randomization work, microbiome signature research, and human intervention trials. However, a more robust body of microbiota–gut–brain axis research is needed, especially through the lens of prevention, intervention, and rehabilitation. With ethical guardrails in place, greater inclusion of at-risk or justice-involved persons in brain science and microbiome research has the potential to transform justice systems for the better.

## 1. Introduction

*“Of course, if it is the bad bacilli that make men murderers and robbers, they should not be punished. A man is not responsible for what his bacilli force him to do. He is acting under physiological distress and might be a good man if he had a better set of microbes in him.”*

Editorial, “The Bacillus of Crime”—The Springfield Daily Leader, 1910 [1]

The quote above, from a Mid-Western USA newspaper, used sarcasm to mock early 20th century ideas on the biosocial origins of criminal behavior. The editorial positioned the idea of criminogenic microbes as preposterous and used it to scoff at the ‘sentimentalists’ who defended criminals. The editorial did not occur in a vacuum—it was one among several that pilloried the notion that a “Bacillus criminalis” might be discovered [2]. It was written at a time when more than a few physicians and scientists were arguing that gut microbiota were involved in neuropsychiatric diseases [3]. Autointoxication theory posited that disturbances in gut bacteria result in the production and systemic absorption of a variety of putrefactive chemicals, the consequences of which include cognitive and behavioral disruption [4].

Although a minority view, some prominent physicians argued that the consequences of autointoxication extended to criminal behavior: “*The relation of bacteria to vice and crime is not absurdly far-fetched*...*the predisposition* [to crime] *existing, autointoxication does the rest*” [5]. The neuropsychiatric aftereffects of autointoxication were said to be “*a much more frequent cause of serious crime than is generally imagined*” [6]. Dysbiosis-associated autointoxication, at least in some cases, was said to diminish inhibition and promote aggressive tendencies: “*the* [autointoxication] *patient loses control, and fits of irritability or violent passion are not infrequent*” [7]. Some claimed that if gut dysbiosis and autointoxication were to be scientifically validated, then “*crime will one day be shown more definitely than ever to be a matter dealt with by medical science rather than by law*” [5]. It was reported that researchers from Pasteur’s laboratory observed hoarding and purloining among animals injected with a bacterium dubbed “Bacillus of Kleptomania” [8]. Writing in the journal *Mind* in 1897, one author anticipated chaos in the courts: “*For what in the world shall we do if an illogical public gets the idea that micrococci and bacilli are the cause of human faults and frailties? We shall soon have the expert in our courts discoursing of a Kleptococcus*” [9].

More than a century on, the idea of gut microorganisms as part of a criminal defense, sentencing mitigation strategy, and/or a detention/parole neuroprediction composite, may seem no less fantastical. However, advances in neuromicrobiology, along with related omics technologies, are lending weight to the idea that gut microorganisms contribute to aggressive and/or antisocial behavior. Indeed, recent court decisions in the United States and Europe have demonstrated—at least in the case of auto-brewery syndrome—that gut microbes are considered ‘responsible’ for what would otherwise be criminal behavior.

In this Viewpoint article, we will discuss the auto-brewery syndrome decisions, arguing that they warrant a much larger incorporation of neuromicrobiology and multi-omics science within the criminal justice system, as well as mental health treatment of aggressive or violent behavior. We explore the potential mechanisms of action that connect gut microbiota to cognition and behavior. Here, we emphasize that these links are not restricted to bacteria alone, and include the trillions of microorganisms—viruses, archaea, fungi, and bacteria—found within the gastrointestinal tract and various ecological niches that make up the microbiome. The latter term describes both the microorganisms and their theater of activity [10]. While ours is a viewpoint article with an emphasis on historical context, and not a formal scoping review, we did identify important articles through the PsycINFO, Google Scholar, and PubMed databases. We supplemented the material found in academic databases with relevant articles drawn from media databases, including *NewsBank* and Ancestry’s Newspapers.com.

At the outset, we note that the term legalome refers to the application of gut microbiota and brain/behavior sciences, including related omics technologies, in the context of forensic neuropsychiatry and legal psychology. As we will show, legalomic science is beginning to challenge prescientific courtroom ideologies of free moral agency and willpower. The emergent research should, at the very least, provoke robust debate and discourse concerning the questions raised by advances in legalome sciences. We are hopeful that our discussion will inspire further research by experts within the interdisciplinary field of brain sciences.

## 2. Auto-Brewery Syndrome

Alcohol has long been recognized as one of the many chemicals manufactured by gut microorganisms. Although the early 20th century autointoxication discourse focused largely on the effects of microbe-produced indole, skatole, p-cresol, and other so-called putrefactive chemicals [9], there was some discussion of internal ethanol (EtOH) production. Mostly, the production of alcohol via the fermentative action of gut microbes (acting on dietary carbohydrates) was considered to be minimal and of little clinical interest. At the margins of medicine, some authors argued that in certain subjects “*the alcohol produced by it* [gut microbe action on carbohydrates] *can be carried to such a degree that a state of chronic alcoholic autointoxication may possibly result*” [11]. Similarly, others claimed that for people with high-sugar, diet-induced dysbiosis, the gastrointestinal tract resembles “*a beer vat at a brewery or a mash tub at a distillery*,” further postulating that this chronic low-grade production of alcohol damages the liver and contributes to sugar cravings [12].

In 1959, the case of Kozo Ohishi, a 46-year-old Japanese man who complained of post-prandial inebriation (absent any alcohol consumption), received significant international media attention. After years of being dismissed with disbelief, Ohishi was tested in a university laboratory setting; the results demonstrated significant elevations in blood alcohol after carbohydrate test meals, and the investigators also reported evidence of *Candida albicans* overgrowth [13,14]. Mostly, the case was treated as a newsworthy medical anomaly. In the decades that followed, sporadic cases were presented in Japanese medical literature [15], and at least one case in the United States received considerable media attention in the 1970s [16]. Dubbed auto-brewery syndrome (or gut fermentation syndrome), it was reported that the rise in blood alcohol in these cases could be associated with irritability, hostility, and even violence [17].

**Figure 1:**
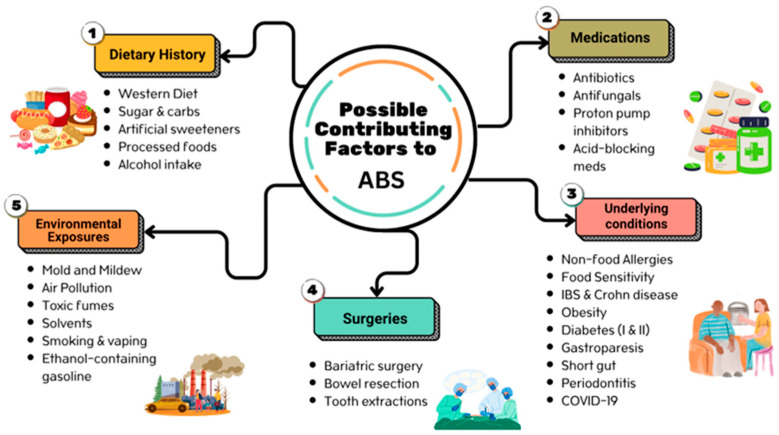
Multiple factors have been linked to auto-brewery syndrome.

Recent years have witnessed a rise in documented cases of auto-brewery syndrome. Although this might be attributed to increased awareness, there are reasons to suspect that dysbiotic drift may be driving higher rates of auto-brewery syndrome. Dysbiotic drift is a term used to describe how modern lifestyles push gut microbial disturbances in non-random ways [18,19]. Well-known dysbiotic forces could include antibiotic overuse, diets dominated by ultra-processed foods, over-the-counter and prescription acid-blocking agents, chronic low-grade chemical exposures, and nutritional inadequacies. These factors, especially antibiotic consumption, have been linked to the onset of various cases of auto-brewery syndrome [20]. Recently, a case of post-COVID-19 infection auto-brewery syndrome (including aggressive behavior) was reported, raising the possibility that COVID-related gut microbiome disturbances increase risk [21] (See Figure 1).

Historically, rare cases of auto-brewery were assumed to be driven by alcohol production by intestinal yeast-like fungi, most notably *Candida albicans* [22]. Since many cases of auto-brewery syndrome appeared subsequent to antibiotic use, a known contributor to *Candida* spp. overgrowth [23], this is a reasonable mechanistic pathway. However, contemporary research shows that various bacterial spp., especially *Klebsiella pneumoniae*, are well capable of producing significant amounts of alcohol [24,25]. Like *Candida* spp., *Klebsiella pneumoniae* growth is encouraged by simple sugars in the diet [26], and overgrowth can occur when exposed to antibiotics [27]. While a full catalog of ethanol-producing microbes found in the human gastrointestinal tract remains to be completed, at least 85 different microbes have been identified, including 61 bacterial and 24 yeast species [24].

## 3. Legalomics and Involuntary Intoxication

To the best of our knowledge, the first attempt to use auto-brewery syndrome in a ‘driving while intoxicated’ (DWI) defense was *State v. Vargas* (1990) in New Mexico [28]. Although unsuccessful—the prosecutor referred to it as “laughable” and the judge waived it off as without “credence” [29,30]—the defense attorney Ray Twohig was prophetic: “*Those who might otherwise be wrongly convicted now have an explanation which may someday be better researched and understood by scientists, and may provide a defense*” [31]. Indeed, recent years have witnessed DWI dismissals in the United States (2015, 2017) and Europe (2024)—the courts seemingly satisfied with controlled laboratory evidence demonstrating significant elevations in post-prandial blood and breath alcohol levels after carbohydrate test meals [32,33].

These decisions by the court are an acknowledgement that gut microbes are interacting with dietary carbohydrates to produce a mind-altering substance, in this case, EtOH. That is, for the first-time defendant, unaware of a dysbiotic microbiome, they are the victim of involuntary intoxication and are therefore not ‘blameworthy.’ This has cracked open the door to legal questions that extend well beyond the culpability of operating machinery with blood alcohol concentrations (BAC) above jurisdictional limits. Before discussing BAC in more detail, it is worth pointing out that *C. albicans*, the yeast historically associated with auto-brewery syndrome, has also been linked to the severity of symptoms in schizophrenia and bipolar disorder [34,35].

Cognitive changes occur at levels far below the 0.08% BAC limit in the USA and many other jurisdictions (this is why Albania sets their BAC limit at 0.01%) [36,37,38]. Auto-brewery syndrome, a condition that almost certainly sits on a continuum of post-prandial BAC levels, forces broader questions of involuntary intoxication. Moving up the BAC ladder from 0.01% to the 0.08% level may increase risk-taking [39,40] and (depending on personality and provocation context [41]) the odds of altercations [42].

Alcohol is certainly a substance which the defendant knows (or ought to know) can cause intoxication when introduced into the body, and is a substance that is familiar to judges and juries. In this way, auto-brewery syndrome is a relatively easy point of entry for microbial discussions in the courts. Given that *C. albicans* and other microbes influence many brain-related pathways [43], we will raise the possibility of other mind-altering (“intoxicating”) metabolites produced by gut microbes. We emphasize that if auto-brewery is more common than currently assumed, it also forces discussions of potential vulnerability to conflict and/or crime victimization. While blood alcohol is a well-known risk factor for criminal behavior [44], it is also associated with higher rates of victimization [45]. For example, in a US study drawing from the National Violent Death Reporting System, 39.9% of homicide victims had a positive BAC, including 13.7% with a BAC at the lower end, from 0.01 to 0.079% [46]. Similarly, approximately 15% of auto collision fatalities in the US involve BAC levels below 0.08% [47].

## 4. Neuromicrobiology—Potential Mechanisms

Auto-brewery syndrome involves relatively uncomplicated mechanistic pathways from EtOH-producing microorganisms, fermentation, systemic absorption of EtOH, to ease of passage through the blood–brain barrier (BBB). The resultant changes in cognition and behavior are (or should be) understandable to the courts. This is probably why the prosecutor in one of the DWI dismissal cases—who initially vowed to appeal the judge’s decision and reinstate charges—conceded that the objective laboratory results (carbohydrate test meals) are difficult to challenge: “*We have no legitimate grounds we can raise in good faith that would compel the appellate court to reverse*” [48].

**Figure 2:**
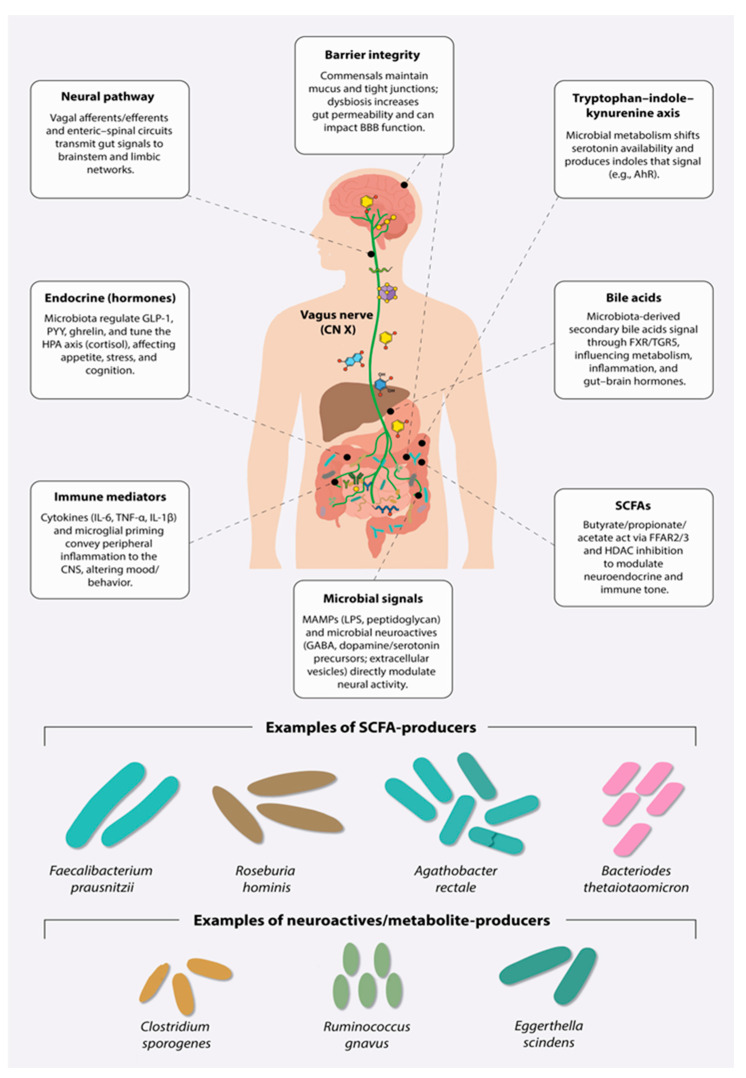
Microbiota-Gut–Brain Communication: Multiple direct and indirect mechanistic pathways have been identified by international, transdisciplinary research.

While auto-brewery provides mechanistic learnings—the relative simplicity of which can assist in adoption of the legalome—mechanistic pathways linking gut microbes to potential criminal behaviors are complex. Indeed, after two decades of intense scrutiny, dating back to the early 2000s [49], the precise direct and indirect forms of communication between gut microbes and the brain are not fully understood. However, there have been remarkable advances in understanding gut microbe-brain connections. What is clear is that there are multiple ways in which gut microbes can interact with and influence host cells. For now, the mechanisms—some very well replicated by international scientists, others emergent—can be described along several distinct but interrelated routes. Readers of *Brain Sciences* may be familiar with these pathways, but they are included here and represented in Figure 2 for the benefit of interdisciplinary scholars.

**Neural pathways:** Several neural pathways are involved in bidirectional communication between the brain and gut microbes: the vagus nerve and spinal afferents serve as conduits through which microbial signals influence brain circuits implicated in emotion regulation, stress reactivity, and executive function [50]. Experimental vagotomy studies in rodents, for example, have demonstrated that certain probiotic strains (e.g., *Lactobacillus rhamnosus* JB-1) lose their anxiolytic or antidepressant effects when vagal signaling is disrupted [51,52]. Gut bacteria are also actively involved in the synthesis and degradation of neuroactive compounds, including gamma-aminobutyric acid (GABA), serotonin precursors, cortisol, short-chain fatty acids, and a range of other metabolites—many of which are capable of crossing the blood–brain barrier and modulating neural activity directly [53,54]. At the molecular level, emerging evidence suggests that microbial activity may shape host epigenetic regulation [55,56] and influence protein glycosylation in the brain, with potential implications for neurodevelopment, synaptic plasticity and neurodegeneration [57]. These pathways highlight a mechanistic basis for how gut microbes influence brain function and behavior, supported by converging evidence from germ-free animal models, human microbiome–neuroimaging studies and targeted metabolomics.

**Immunity:** In parallel, the microbiome interacts with the immune system, shaping systemic inflammatory responses. Given recent evidence suggesting a causal role of the immune system in neuropsychiatric disorders [58] and criminal behavior [59,60], this pathway opens doors to measurable biomarkers. This includes prospective and genetic evidence linking elevated inflammatory signaling to depression risk (e.g., childhood IL-6/CRP predicting adult depression; IL-6 pathway Mendelian randomization), and associations with aggression-related psychopathology [61]. Microbiota-induced alterations in inflammation can, in turn, influence central nervous system function via circulating cytokines, which are known to affect mood, cognition, and behavior [62]. Causality is supported experimentally: peripheral immune activation alters human mood and corticolimbic activity (typhoid vaccination), and anti-cytokine therapy improves depression in high-inflammation subgroups. Mechanistically, microbial metabolites (e.g., SCFAs) shape systemic immunity by promoting regulatory T cells, thereby tuning inflammatory tone that feeds forward to brain function [63,64]. Biomarker candidates include CRP, IL-6, TNF-α, and the kynurenine/tryptophan ratio, which capture immune–metabolic signaling relevant to brain outcomes.

**Metabolism and Endocrine:** Gut microbes support host metabolic and endocrine processes that are tightly coupled to brain health. They contribute to nutrient handling and bioactivation, including microbial production of menaquinones (vitamin K_2_) and conversion of polyphenols (e.g., ellagitannins → urolithins), which alter bioavailability and signaling [65,66,67,68]. Microbiota also shape glucose and lipid homeostasis: colon-derived SCFAs and other metabolites influence hepatic/adipose metabolism and insulin sensitivity; mechanistic and translational evidence includes (i) lean-donor FMT improving insulin sensitivity in metabolic syndrome patients, and (ii) *Akkermansia muciniphila* improving metabolic profiles [69,70]. Enteroendocrine cells (EECs), hormone-producing cells located in the epithelial layer throughout the gastrointestinal tract, are responsive to gut microbes. Given that EECs have neuronal-like characteristics, acting as a bridge to enteric nerves and the central nervous system, their role in schizophrenia and other neuropsychiatric conditions is under increased scrutiny [71]. At the gut–endocrine interface, SCFAs activate FFAR2/FFAR3 on enteroendocrine L-cells, increasing GLP-1/PYY and modulating appetite and energy balance. The microbiome also remodels the bile-acid pool via deconjugation and 7α-dehydroxylation, generating secondary bile acids that signal through FXR and TGR5 to regulate glucose/lipid metabolism and gut–brain endocrine pathways [72,73]. These nutrient, SCFA, enteroendocrine and bile-acid routes provide plausible endocrine mechanisms linking diet–microbe interactions to central function via hormonal, vagal and immune crosstalk.

**Gut Health:** Moreover, under homeostatic conditions, gut microbes help maintain the structural and functional integrity of the intestinal barrier. Mechanistically, commensals and their short-chain fatty acids (especially butyrate) support mucus-layer renewal and tighten epithelial junctions (occludin/claudins/ZO-1), thereby sustaining barrier function [74,75]. However, microbial dysbiosis can compromise this barrier, leading to increased intestinal permeability—often referred to as “leaky gut”—via mucus erosion during low-fiber states and tight-junction dysregulation; these changes are linked to systemic “metabolic endotoxemia” (circulating LPS) and low-grade inflammation [76]. Barrier failure in the gut is also associated with altered blood–brain barrier (BBB) integrity: germ-free mice show increased BBB permeability that is normalized by colonization or SCFAs, and systemic inflammatory challenges (e.g., LPS) disrupt BBB tight junctions and increase tracer leakage [77,78,79]. Such breaches may allow translocation of microbes and/or microbial products (e.g., LPS, peptidoglycan) and peripheral inflammatory signals to influence central function, potentially contributing to changes in mood, cognition and behavior [80,81]. Of course, ‘gut health’ is a broad term with different interpretations. One unifying principle is that gut health (loosely defined as ideally maintained structure/function with optimal microbial composition) cannot be uncoupled from lifestyle, environmental exposures, and socioeconomic forces. This will be explored further in Section 7 below.

## 5. Microbial Signatures and Multi-Omics Support

In concert with mechanistic studies, the last two decades have witnessed numerous preclinical and human population studies linking disturbances of the gut microbiome to a wide variety of neuropsychiatric disorders. Since mental disorders among justice-involved and incarcerated people are higher than in the general population [82,83], these studies are, in effect, an examination of a potential forensic pipeline [84]. Attention-deficit hyperactivity disorder, conduct disorder, posttraumatic stress disorder, or schizophrenia—all have been associated with microbiome disturbances [85,86,87,88], and all are associated with significantly higher risks of involvement with the legal system [89,90,91,92].

Connected to these efforts is the identification of gut microbial signatures that reflect personality [93] and/or represent aspects of cognition and behavior that might otherwise be associated with higher or lower risks of justice involvement. For example, human gut microbial signatures have been linked to the regulation of emotions [94,95], stress resiliency [96], temperament [97,98], empathy/compassion [99], impulsivity [100,101], reactive aggression [102], self-harm [103], and aggressive or violent tendencies [104,105]. Researchers are exploring the directionality of the relationships between impulsivity, sensation-seeking, and microbial signatures, and how these might intersect with environmental factors such as dietary patterns [106].

These advances represent a move beyond mere signs of ‘dysbiosis’ to microbial signatures that hold diagnostic promise. Recent evidence indicates that microbial signatures have the potential to identify persons living with schizophrenia or bipolar disorder [107], autism spectrum disorders [108], or ADHD [109]. For example, the genera *Eggerthella* and *Lachnoclostridium* is enriched in bipolar and schizophrenia spectrum disorders compared to healthy controls [107]. Antisocial behavior among youth is a predictor of suicidal behaviors [110] and emergent research shows that people who attempt suicide have distinct microbiota signatures (e.g., higher levels of *Fenollaria timonensis* and lower levels of *Corynebacterium aurimucosum*) [111]. While the ‘ideal’ microbiome will likely remain elusive [112], researchers are moving closer to microbial profiles that help identify people with mental disorders in general, compared to unaffected individuals [113]. 

In the context of diagnostics and risk identification, advances in omics technologies allow researchers to match microbial signatures with biological markers (i.e., metabolomics, genomics, epigenomics, and transcriptomics) and aspects of cognition and behavior [114,115]. While auto-brewery is focused on one gut microbial-produced chemical—EtOH—metabolomics helps identify a massive array of gut microbe-produced metabolites. Aided by investments in large cohort studies and machine learning, researchers use quantitative biomolecular readouts (via blood, saliva, fecal samples, and/or exhaled breath) to accurately identify chemicals related to mood and behavior [116]. Even more impressively, researchers can now collate microbial signatures with polygenic risk scores and measurable metabolites, allowing for a more robust composite of risk for aggression or justice involvement [117].

In an interesting twist, several of the gut microbial metabolites once claimed to be associated with neuropsychiatric conditions during the early 20th-century autointoxication era are now being reidentified with risk. For example, gut-derived p-cresol has been linked to dopaminergic disruption in the brain [118]. Elevated levels of propionic acid (a gut microbe-produced short-chain fatty acid) are linked to cognitive decline [119], aggression and disturbances in social behavior [120], and changes in brain synaptic architecture [121].

Even though genetic factors are estimated to account for some 40 to 60% of the variance in aggressive, antisocial, and criminal behavior [122,123,124], the quest to tie single genes to justice involvement has been disappointing [125]. Polygenic risk scores have the potential to improve on this, helping to identify behavioral risks, for example, in the area of lack of self-control [126]. Omics-based technologies illuminate pathways of gene expression, which leads to better mechanistic linkages between environmental exposures and the regulation of gene expression [127]. The promise of omics technologies and epigenetics (including that mediated by the microbiome [128,129] extends to the prevention of criminal behavior and personalized treatment/rehabilitation of justice-involved persons [130,131]. In addition, these technologies represent a way to link isolated ‘lifestyle’ risk factors (e.g., ultra-processed food consumption) with metabolomic and microbiome signatures [132]. For example, researchers identified blood and urinary molecules that accurately reflect how much of a person’s diet includes ultra-processed foods [133]. These efforts will help to close the gap between correlation and causation [134], a topic we turn to next.

## 6. Are Gut Microbes a Causal Factor?

The bulk of research in the area of neuromicrobiology and nutritional criminology remains in preclinical and epidemiological realms, with a small but growing number of clinical intervention trials. Notwithstanding auto-brewery syndrome, where microbial dysregulation contributes to aberrant behavior, establishing a causal link between gut microbiota and criminal behavior is an inherently complex challenge. However, at least one compelling line of investigation is strengthening the argument for causation—fecal microbiota transplantation (FMT), wherein gut microbial communities from one organism are transferred to another.

FMT experiments are commonly performed in animal models, where ‘donor’ animals—either healthy or exposed to specific stressors such as high-fat diets, social isolation, or chronic stress—serve as the microbial source for ‘recipient’ animals [135]. In some cases, the donors are human, currently diagnosed with conditions like depression, autism spectrum disorder, or substance use disorders, with their gut microbiota transplanted into healthy rodents. Following transplantation, recipient animals often exhibit rapid and significant shifts in behavior and physiology. When the microbial donors have histories of neuropsychiatric disorders, recipient animals begin to mirror behavioral and cognitive characteristics typical of the human condition, such as heightened anxiety, altered mood, cognitive impairment, or signs of dependency [136,137,138,139,140,141]. Human research has connected early life antibiotic exposure to subsequent behavioral problems [142]; fecal transplant from children exposed to early life antibiotics increases aggression in recipient animals [143]. The behavioral changes are notably absent when the transplanted material is derived from psychologically healthy individuals or those who did not experience the environmental variable in question.

Some of the FMT research has examined the intersection of microbiota and behavioral phenotype. For example, when BALB/c mice (a strain known to be highly sensitive and timid) were the recipients of intestinal microbes from NIH Swiss mice (a strain known to be less timid and more explorative), the typically anxious BALB/c mice became more explorative. The opposite occurred when NIH Swiss mice were colonized with BALB/c microbiota [144]. Similarly, gut microbiota sourced from animals accustomed to chronic alcohol exposure can induce anxiety- and depression-like behaviors in healthy recipients [145]. On the other hand, FMT from healthy animals appears to curb drug addiction-related behaviors and withdrawal symptoms [146]. Similarly, when animals that experienced early-life trauma receive microbiota from healthy donors, improvements in cognitive performance have been noted [147].

These behavioral transformations are increasingly supported by measurable biological changes. Animal recipients of ‘dysbiotic’ microbiota show alterations in gene expression within the brain, elevated levels of inflammation in both the gut and central nervous system, weakened blood–brain barrier integrity, disruptions in neurotrophic signaling, and disturbances to serotonergic or gamma-aminobutyric acid (GABA) neurotransmitter systems [148,149]. The transfer of bacteriophages can also induce changes. For example, animals receiving fecal microbiota and viral transplantation from human donors with the highest Gokushovirus load exhibit addiction-like behaviors. These behavioral disturbances were accompanied by alterations in dopamine receptors, and changes in brain tryptophan, serotonin, and dopamine metabolism [150]. Related to auto-brewery syndrome, it is also worth noting that in animal models FMT sourced from healthy specific pathogen-free donors prevents colonization of *C. albicans* [151].

Taken together, these preclinical transplant findings suggest that microbial ecosystems in the gut are not only capable of influencing mental states and behavioral outcomes—they may also transmit vulnerability [152,153,154]. The implications are profound, pointing toward the microbial transmissibility of cognition and behavior that otherwise increases the odds of justice system involvement [155]. Remarkably, a small but growing number of human studies—including case reports and pilot trials—are beginning to validate the preclinical findings. Early evidence supports beneficial effects of FMT in overall mental health [156,157], autism [158], depression [159], aggression [160], insomnia [161], and alcohol use disorder [162]—in the latter case, reducing craving and consumption. At least two case reports have documented the resolution of auto-brewery syndrome following fecal transplant [163,164]. Encouragingly, researchers have reported long-term benefits in metabolic markers in young people with obesity, even four years after the original FMT procedure [165].

In addition to FMT studies, Mendelian randomization (MR) studies are also pointing in the direction of a causal role for gut microbiota [166,167]. MR uses genetic variants as instrument variables to tease out and assess the causality of modifiable exposures to outcomes. For example, recent MR research has identified bacterial taxa that appear to have causal roles in conduct disorder [168], schizophrenia [169], bipolar disorder [170], and addiction disorders [171,172], conditions that represent major risks for justice involvement.

In further support for a causal role, a small but growing number of intervention studies demonstrate that oral probiotics (and related agents capable of targeting the gut microbiome) can influence justice-relevant behaviors. For example, human studies show that these agents can reduce aggression [173,174], aggressive thoughts [175], impulsivity [176,177,178], anger [179], irritability [180], negative mood [181], risk-taking [182,183], and enhance emotion regulation [184] and accuracy in identifying faces expressing various emotions [185]. However, it is important to note that most trials are small, short, or protocol-stage, so effect sizes and durability remain uncertain and need preregistered, adequately powered replication.

The ‘holobiont blindspot’ captures a deeper issue: by treating the human as a host-only unit, we miss the co-acting microbial partners that help shape cognition, affect and behavior. The holobiont concept (host + microbiome as an interacting unit) and the hologenome framework argue that host traits emerge from host–microbe consortia, so host-only models risk systematic bias in both explanation and intervention design [186]. This blindspot has been explicitly articulated in psychology and behavior science, proposing that overlooking host–microbe influences is a cognitive bias with real-world ramifications for social and justice outcomes [187]. Methodologically, closing the blindspot means co-phenotyping hosts and microbes: integrating longitudinal metagenomics/metabolomics with host genomics, immune and endocrine readouts (e.g., SCFAs, bile acids, cytokines), and preregistered RCTs that couple behavioral endpoints with microbial mechanisms (strain-level, metabolite-level) and causal triangulation (MR <-> trials <-> natural experiments). Recognizing and addressing this blindspot could ultimately inform fairer, more evidence-based approaches within the legal system, where understanding (micro)biological contributors to behavior is critical.

## 7. Match to Existing Biopsychosocial Research

Volumes of international research demonstrate that behaviors otherwise associated with justice involvement (e.g., aggressive, violent, antisocial, and deceptive actions) have complex biopsychosocial antecedents. In particular, socioeconomic marginalization [188], inequality [189], and adverse childhood experiences (ACEs) [190] are consistently associated with higher risks of justice involvement. Arrests and incarcerations can compound existing social marginalization and material deprivation, leading to further socioeconomic health disparities [191,192].

While researchers have yet to scrutinize microbiome markers as specific indicators of justice involvement risk, the existing research on socioeconomic disadvantage and the gut microbiome supports a ‘dysbiotic drift’ theory [18]. That is, marginalization and deprivation in industrialized countries are associated with environmental exposures (or so-called ‘lifestyle’ factors) that lead to dysbiosis. These factors might include food insecurity and related risks of higher ultra-processed food consumption, environmental toxins, heavy metals, and a host of other exposures that do not sit on a level socioeconomic playing field [18].

Importantly, there are intersections between these socioeconomic factors. For example, the combination of bisphenol A exposure and an unhealthy dietary pattern, both of which are more likely according to disadvantage, leads to dysbiotic changes and worse behavioral outcomes [193]. Indeed, the available research points toward socioeconomic deprivation as a factor in dysbiosis [194,195,196], and once in place, the altered microbiome compounds the risk of neuropsychiatric disorders [19].

In a recent dissertation study, census block crime data were paired with post-mortem gut microbiome samples. The results showed that the alpha diversity of the gut bacteria (richness and evenness) was significantly lower in areas with high reported crime. *Lachnospiraceae*, an anaerobic bacterial family associated with neuropsychiatric conditions and aggression, was specifically linked to area crime levels [197]. ACEs have also been linked to dysbiosis. For example, ACEs have been reported to mediate associations between dysbiosis (i.e., compositional and functional dysbiosis, marked by depletion of beneficial taxa (e.g., *Bifidobacterium*, *Faecalibacterium*) and enrichment of pro-inflammatory genera (e.g., *Escherichia-Shigella*)) in young people with depression [198].

Emergent studies also indicate that there are differences in the gut microbiome in people who are in carceral settings. For example, researchers in the Czech Republic reported that female prison inmates with a history of violence and impulsivity (compared to healthy controls from the community) have a distinct gut microbiome profile, with higher levels of the genera *Bacteroides*, *Barnesiella* and the order *Rhodospirillales* [199]. In a small study (*n* = 31), Chinese researchers found that compared to healthy non-incarcerated controls, prisoners recently admitted to two jails (i.e., samples retrieved within one month of admission) had significant differences in the phylogenetic structure and functional genes of the gut microbiota [200]. As discussed in the Future Directions section below, there is a need to determine if the conditions of confinement (e.g., diet, stress, isolation, hygiene) provide explanatory power, or whether individual differences in gut microbes can explain behavior in carceral settings (or both).

## 8. Response to Glucose Testing and Brain Fog

Researchers and clinicians query auto-brewery syndrome by administering large amounts of glucose and a carbohydrate meal while the subject is in a fasted state. For example, in the case of post-COVID auto-brewery syndrome mentioned earlier, the researchers administered 200 g of glucose and then measured BAC in the postprandial phase [21]. We suspect that glucose and other carbohydrate testing offers an opportunity for justice-related gut–brain-behavior discoveries that can extend beyond auto-brewery syndrome.

Large numbers of gastroenterology clinic patients report neuropsychiatric symptoms, and the opposite is also common—gastrointestinal complaints are frequently reported in neuropsychiatric patient populations. If there is an absence of evidence indicating ‘structural’ tissue damage, the patients with gastrointestinal complaints are said to be suffering from disorders of gut–brain interaction (DGBI). Research shows that an oral glucose intake of 75 g can provoke a variety of gastrointestinal symptoms in adults with DGBI [201]. For the discussions that follow, it is important to note that brain fog is a common complaint among patients with DBGI [202]. Brain fog as a complaint is associated with irritability and functional impairments in driving [203].

In one 1990 study involving patients in a clinic attending to DGBI complaints, researchers reported that the majority of subjects (over 60%) had significant elevations in blood EtOH levels after an oral glucose load [204]. Several patients had post-glucose blood alcohol levels at meaningful levels, and one subject (after a small 5 g glucose test) had a rise in BAC that matches Sweden’s DWI limit of 0.02% [205]. In a similar study involving patients with DGBI symptoms, researchers using a low-dose glucose test found a wide range of postprandial blood EtOH levels (6.5-fold difference between lowest and highest subjects) [206]. In a hospital-based study of 75 g intake in nondiabetic adults (*n* = 231, age 40–65 years), 33% of the subjects reported symptoms of dysglycemia, including brain fog [207].

Glucose studies have proved useful in identifying the extent to which people with metabolic dysfunction-associated steatotic liver disease (MASLD, formerly known as nonalcoholic fatty liver disease or NAFLD) are manufacturing EtOH via gut fermentation. Research shows that over 60% of patients with MASLD have high levels of *K. pneumoniae*; after an oral glucose tolerance test, subjects with MASLD had BAC levels 3.6 times higher than healthy controls, and subjects with non-alcoholic steatohepatitis (NASH) had BAC levels 6.5 times higher than controls [208]. In the context of potential justice involvement, it is worth noting that MASLD is associated with significant cognitive impairment [209]. Preclinical MASLD research shows that gut dysbiosis is involved in significant elevation of propionic acid [210], the aforementioned short-chain fatty acid linked to cognitive impairments. Human research supports the dysbiosis-propionic acid connection in liver disease and obesity [211,212].

People with MASLD also have higher serum levels of D-lactate [213], another gut microbe-mediated chemical with neuropsychiatric implications. Brain fog is linked to an overgrowth of D-lactate-producing bacteria in the gut [214,215]. Much like EtOH, an oral glucose load (75 g) appears to be useful in identifying individuals experiencing postprandial elevations in urinary D-lactate coincident with brain fog [214]. Elevated blood and brain levels of lactic acid and lactate (particularly the D-isomer) are linked to neuropsychiatric disorders and aggression [216,217,218]. Research dating back to the 1950s demonstrates that adults with neuropsychiatric conditions—including schizophrenia, psychosis, and bipolar disorder—have significant elevations in lactic acid after glucose or fructose loads [219,220]. Subjects with these conditions also have difficulty metabolizing D-lactate [221]. While more research is needed, is it possible to sequester D-lactate in the gut to prevent MASLD and its associated symptoms [222,223]?

Taken together, the available evidence suggests that there are multiple opportunities to perform research-based oral glucose tolerance tests in various patient populations, with the aim of assessing subsequent elevations in EtOH, D-lactate, or other microbe-mediated chemicals. As many as 40% of adults in the US suffer from DGBI [224], and as many as 32% have MASLD [225]. If even a small percentage of these populations are unknowingly manufacturing significant levels of internal EtOH and/or D-lactate, it would equate to significant risks of justice involvement. Moving toward a fine-grained study of DGBI and MASLD should be part of future research directions.

## 9. New Bottles for Old Wine

Advances in neuromicrobiology and omics allow for a revisit of topics that are poorly understood, including those that represent high risks for justice involvement. For example, it is estimated that some 30–40% of patients with depression experience anger attacks—sudden onset anger accompanied by intense symptoms of autonomic activation. Anger attacks include rapid-onset, unplanned thoughts of attacking others, physically or verbally, and throwing or destroying objects [226,227]. Despite the physiological resemblance between anger and panic attacks, fear is absent or less pronounced in the former, and anger is generally absent in the latter [228]. Recently, anger attacks have been linked to suicidal ideation [229] and persistent irritability [230]. Despite being an obvious pathway to justice involvement, anger attacks have received little attention in academic criminal justice and forensic psychology.

Among affective personality features in adults with depression, anger is strongly associated with suicidal ideation [231]. Here, the biological differences between suicide by violent (e.g., gunshot, hanging) and nonviolent (e.g., overdose, poisoning) means may be worth examining [232]. Longstanding research has linked suicide by violent means with a prior life course history of impulsivity and aggression [233]. In a recent study, researchers noted significant differences in the gut microbiota of persons who had completed suicide by violent means (i.e., hanging or jumping from a high place) [234]. Given that self-harm and suicidal ideation/attempts can predict subsequent justice involvement, scrutiny of the microbiome represents an important area of research in the realm of prevention science.

Another area that should be approached with renewed vigor is the relationship between parasitic infections and behavioral disturbances. For decades, there have been suggestions that the gastrointestinal pathogen *Toxoplasma gondii* can promote suicidality, risk-taking, aggression, antisocial behavior, impulsivity, self-directed violence, and higher risks of ADHD severity and psychosis [235,236,237,238,239,240,241]. *T. gondii* seropositivity is associated with a higher risk of vehicular accidents [242,243,244,245]. *T. gondii* infection has been shown to disturb the blood–brain barrier, promote neuroinflammation, and disrupt metabolic pathways and neurochemistry in the brain [246,247]. The precise mechanistic pathways between *T. gondii* infection and neuropsychiatric disturbances remain obscure, although they appear to be mediated by *T. gondii*-induced gut dysbiosis and intestinal permeability [248,249,250]. Fecal transplant research demonstrates that healthy animals experience behavioral disturbances and transcriptomic alterations in the amygdala when in receipt of T. gondii-related dysbiotic fecal material [251].

Another area that warrants attention is the relationship between pediatric acute-onset neuropsychiatric syndrome (PANS) and the gut microbiome. PANS represents a broad spectrum of acute-onset neuropsychiatric symptoms that are oftentimes triggered by microbial infections, including pediatric autoimmune neuropsychiatric disorders associated with streptococcal infections (PANDAS). While PANS is generally recognized by symptoms that reflect obsessive–compulsive disorder, sudden-onset irritability, aggression, and/or severe oppositional behaviors are often part of the presentation [252]. Obviously, the latter symptoms can represent a path to subsequent justice involvement [253]. Although the mechanistic pathways between infection exposure and neurobehavioral symptoms are complex, accumulating evidence points toward infection-associated gut dysbiosis as a contributing factor [254]. Whether or not there are relationships between PANS and later life justice involvement is worthy of scrutiny.

It is also noteworthy that approximately half of the children with PANDAS experience nocturnal enuresis [255]. Nocturnal enuresis has been noted as a risk factor for PTSD [256] and justice involvement [257,258]. Despite being a brain-related condition associated with deficits along neuroinhibitory pathways, enuresis is often erroneously assumed to be a psychological disorder [259]. Recent studies have connected changes in the urobiome to enuresis [260,261]. Historically, aggression and other behavioral problems have been assumed to be a psychological consequence of nocturnal enuresis, rather than being part of central inhibitory deficits that are driven by microbes along the gut-bladder axis [262]. We suspect that dysbiosis is a factor in relationships between enuresis and behaviors that might otherwise lead to justice involvement.

There are many outstanding questions related to pathogenic microbes and behavioral changes that might lead to aggression, violence, and justice involvement. Intriguing research points toward tick-borne infections (e.g., *Borreliaceae* family spirochetes) as contributors to substance abuse, violence, self-harm, and even homicide [263,264,265,266]. This deserves research attention, including a better understanding of the ways in which tick-borne infections might cause behavioral disturbances via induction of gut dysbiosis [267,268].

## 10. Legalome—Future Directions

To date, the field of forensic microbiology has not penetrated the realms of forensic psychiatry and psychology. Current applications of forensic microbiology include evidence that might establish postmortem intervals, aid in human identification, place a perpetrator at a crime scene, and/or help establish a cause of death [269,270,271]. In contrast, the legalome focuses on the potential explanatory power of microbes and microbe-derived metabolites in the context of cognition and behaviors that might otherwise lead to, or be associated with, justice involvement. The legalome includes consideration of the ways in which the human microbiome acts as a biological transducer of multiple environmental inputs. As with the larger frame of legal psychology, the legalome concept also includes the potential influence of microbes in the cognition and behaviors of professionals operating within the criminal justice system—this includes potential connections to lifestyle, workplace performance, excessive use of force, decision-making under stress, and occupational burnout [272].

Until now, the courts have largely kept neurobiological sciences at arm’s length [273]. That is, attempts to use neurosciences, behavioral genetics, or evidence of the long neuropsychiatric reach of childhood trauma/abuse are often rejected by judges and juries when used as a defense or in sentencing mitigation [274,275,276]. Neurolaw, a term that refers to advances in brain sciences that might inform court decisions and policy, including those related to criminal culpability, has remained limited in its reach [277]. However, we argue that linking the gut microbiome and its metabolites with the brain and behavior—through specific causal pathways using multimodal advances in neuromicrobiology and omics technology—will provide a more robust challenge to the ‘status quo’ of the courts.

How, then, can the legalome concept move forward? For microbiome science to influence judicial processes and the criminal justice profession, it must prove its practical value in prevention, treatment, rehabilitation, and occupational wellness. To move the legalome in that direction, there is a need to integrate, improve upon, and translate the (often) siloed mechanistic bench science, epidemiological evidence, and intervention studies. We suggest attention to the following areas:

1. Expansion of preclinical mechanistic research, including a greater linkage between specific biological markers and justice-related behavior. Further identify microbiota-mediated pathways between dietary components and EtOH production [278]. While EtOH is easily understood from a forensics perspective, the courts will need reliable causal links between specific microbes and microbe-manufactured chemicals (e.g., p-cresol, propionic acid [279,280]) and microbe-related chemicals (e.g., plasma pro-inflammatory cytokines [281]). Relationships between elevated uric acid [282] and low bilirubin [283] have been noted in impulsive aggression, and these associations are likely mediated, at least in part, by gut microbiota.
2. Approach criminology with the exposome in mind. That is, consider the biological responses of the “total organism to the total environment” throughout the life course [284]. With advances in exposome science, aided by omics and inclusive of microbiome endpoints, the capacity to examine total lived experiences (both positive and negative “exposures”) interacting with genes (over time) is within reach [285,286]. It is important to consider the potential of microbiomes as contributors to the neurobiology of love and other positive emotions [287].
3. Expansion of high-quality randomized controlled studies, including designs that target the microbiome. With appropriate informed consent and ethical guardrails in place, greater inclusion of at-risk or justice-involved persons in microbiome research. The emergence of microbial signatures related to specific disorders and behaviors requires consideration of vulnerable populations, often excluded from research endeavors. Meaningful inclusion, with consideration of life course experiences and the social exposome, will better inform researchers as they attempt to expand microbiome knowledge [288]. Ethical inclusions of justice-involved persons can help researchers pursue meaningful endeavors, especially those that might improve individualized outcomes.
4. It is important to examine non-violent criminal behavior. Approximately half of the adults in the US jail/prison population are incarcerated for non-violent reasons [289]. So-called white-collar crime is associated with significantly higher rates of recidivism than violent crime [290]. White-collar crime has been linked to genetics and personality traits [291,292]. How might microbial signatures interact with multi-omics and polygenic markers in non-violent populations? How might microbial signatures influence risk-taking in healthy populations [293], and people involved with the justice system?
5. Determine whether or not microbiome or omics-based markers change in response to therapeutic interventions in carceral settings. Does the gut microbiome play a critical role in explaining observed associations between inflammatory diets and behavioral disinhibition [294]? Some US prisons are making efforts to transform their food systems for the better, which represents an opportunity for microbiome and behavior research [295]. Given emerging evidence indicating that baseline microbiome profiles predict antidepressant treatment outcomes [296,297], it might be worth querying whether microbial profiles predict responsiveness to various carceral programs. Meta-analyses of human studies demonstrate the value of various strains and species of probiotics in neuropsychiatric conditions [298,299], yet carceral populations have thus far not been included in this research.
6. Incorporation of a ‘justice lens’ in microbiome research. For example, the phenomenon of ‘brain fog’ is witnessing increased research attention, especially because it is a central complaint associated with rising rates of post-COVID conditions (also known as, ‘long COVID’) [300]. When researchers examine phenomena such as ‘brain fog’ and mental fatigue using multi-omics and machine learning approaches [301], there is an opportunity to consider the risks of justice involvement. Recent population studies have linked adherence to ADHD medications with lower subsequent risk of criminality [302,303]. Given that ADHD medications may (at least in part) operate via the gut microbiome [304], this is an area worthy of scrutiny.
7. Scrutinize the role of the microbiome in the links between trauma exposure and subsequent justice involvement. Military veterans with posttraumatic stress disorder (PTSD) are 61% more likely (vs. veterans without PTSD) to be justice-involved, and the odds of arrest for violent offenses are 59% higher [305]. Research shows that the gut microbiome is disturbed in PTSD, with one recent study linking dysbiosis to executive function in veterans with PTSD [306]. Given the long-standing research connecting deficits in executive function to significantly higher risks of justice involvement [307], this is an area worthy of pursuit. Since gut microbes appear to mediate the startle response [308], deficits of which have been found in PTSD and violent/antisocial behavior [309,310,311], this is an area ripe for research.
8. Revisit existing research with legalomics in mind. For example, multiple studies have connected the quantity and accessibility of residential greenspace with positive mental health, and even reduced violence and crime [312,313]. The mechanisms are poorly understood, but are often assumed to operate through sensory pathways leading to cognitive restoration and stress reduction. However, emerging research is connecting environmental exposures, including greenspace, to differences in gut microbes [314] and temperament [315]. Moreover, emerging research has linked skin microbiota to psychological wellbeing [316].
9. Expand the use of neuroimaging and electroencephalogram research in concert with omics, microbial signatures, and interventions targeting the microbiome. For now, only a small number of studies have incorporated neuroimaging and EEG into intervention trials [317] or research examining microbiome signatures with brain areas involved in with memory, language, and emotion processing [318].
10. Explore how neuromicrobiology and metabolomic markers can enhance the predictive value of currently used “paper and pencil” instruments used in parole and probation risk assessments. Viewed through a lens of diagnostic accuracy in health and medicine, commonly used criminogenic risk assessments would be rated as “poor” or “fair”—they may be enhanced significantly by supplementing with biological markers [319].
11. Enhance education, scientific literacy, and develop ethical frameworks. Legalome research—linking microbiology to legal responsibility—raises fundamental questions about punishment. Studies show that many lawyers and judges lack formal scientific training and rely heavily on personal beliefs [320]. Research has found that potential jurors with higher levels of scientific knowledge are less likely to support harsh sentencing [321]. Continuing education for the legal community, encompassing advances in microbiology and gut microbiome, should be explored. Education and science literacy will be essential to science-based reforms. Building bridges between microbiology and justice will require new frameworks, deeper collaborations, and cultural shifts in how responsibility, punishment, and public safety are understood. Matters of privacy and the risks of “dangerousness” labeling will need to be considered [322]. How might potential biases or misinterpretations in the analysis of gut–brain axis findings lead to potential harms to an individual and society?
12. Establish an epidemiological understanding of auto-brewery syndrome and explore connections to mental health and suicidality [323]. While there are multiple reasons to suspect that the condition is more common than currently appreciated, the frequency of the condition remains a matter of speculation. Cases are typically uncovered and reported only when the BAC is exceedingly high [324].
13. Explore short-term changes in cognition and behavior after single or several meals. There is evidence to suggest that meals rich in ultra-processed or fast foods can lead to changes in post-prandial physiology [325], cognition [326], and the microbiome transcriptome [327]. How might this influence the “irresistible impulse” in criminal law or the rapid decisions made by criminal justice professionals?
14. Begin to examine relationships between the lifestyles of criminal justice professionals, occupational performance, and objective microbiome-related markers. For many in law enforcement, an inflammatory diet may be the norm [328,329]. Recent evidence suggests that many correction officers may be underpaid and living with food insecurity [330]. Given links between food insecurity and higher consumption of ultra-processed foods [331], and alterations in the microbiome [332], there may be microbiome-mediated behavioral changes in the workplace.
15. Cross-jurisdictional data integration. Synchronize microbiome, behavioral and legal outcome datasets across countries and legal systems to understand how cultural, dietary, environmental, and policy differences modulate microbiome–justice relationships. Bioinformatics pipelines, including microbiota analysis for personalized care, at a nascent stage. Per the recent Position statement of the Microbiota International Clinical Society, “explicit reporting of software and database releases, key parameters and their rationale, and, where feasible, simple sensitivity checks to confirm the robustness of main findings” are recommended [333].

## 11. Conclusions

Interest in microbes and biological factors as contributors to criminal behavior waxed and waned throughout the 20th century, with renewed attentiveness in recent years. Using auto-brewery syndrome as a central example, we highlighted multiple research studies in humans that demonstrate linkages between the gut microbiome and the brain. Gut microbes communicate back and forth with the brain using multiple and complex pathways, including immunological and metabolic enzymes, metabolites, and other microbe-mediated chemicals. Gut microbes contribute to the synthesis of neuroactive agents and inflammatory responses implicated in neuropsychiatric disorders and criminal behavior.

Fecal transplants, probiotic studies, and Mendelian randomization research further support the idea of a causal role of the gut microbiome that connects biopsychosocial factors, gut dysbiosis, and justice involvement. The bulk of the research sits in preclinical models and exploratory stages. As outlined in the Future Directions Section, there is much work to be performed in establishing a strong evidence base around the legalome concept. However, researchers are moving toward the mapping of microbial signatures that can be linked to various behaviors, including aggressive or violent tendencies. Viewing this microbiome-related body of research supports the legalome idea and challenges courtroom tenets of free moral agency and willpower.

We have argued that experts in neuromicrobiology, neuropsychiatry, and allied sciences, have the potential to further our understanding of criminal behavior, a complex and enduring biopsychosocial phenomenon. Given the massive costs and public health consequences of crime [334,335], investments in research should be considered urgent. We are hopeful that our treatise will inspire directions for transdisciplinary research applicable to criminal justice.

## Data Availability

No new data were created.

## Abbreviations

The following abbreviations are used in this manuscript:

ABS	Auto-brewery syndrome
AhR	Aryl Hydrocarbon Receptor
BBB	Blood–brain barrier
MAMPs	Microbial-associated molecular patterns
